# *Streptococcus intermedius* cerebellar abscess as first presentation of sarcoidosis: a case report

**DOI:** 10.1186/s13256-026-06095-8

**Published:** 2026-05-25

**Authors:** Abdulrahman Al-Ahmadi, Anatoli Pinchuk, Klaus-Peter Stein, Belal Neyazi, Andreas E. Zautner, Achim J. Kaasch, Ibrahim Erol Sandalcioglu, Ali Rashidi

**Affiliations:** 1 Department of Neurosurgery, Otto Von Guericke University, Magdeburg, Germany; 2 Institute of Medical Microbiology and Hospital Hygiene, Medical Faculty of the Otto Von Guericke University, Magdeburg, Germany; 3 Department of Laboratory Medicine and Microbiology & Vaccination Center, Klinikum Würzburg Mitte gGmbH, Würzburg, Germany

**Keywords:** *Streptococcus intermedius*, Cerebellar abscess, Sarcoidosis, Central nervous system infection, Immunosuppression, Case report

## Abstract

**Background:**

Cerebellar abscesses are rare but potentially life-threatening infections that require prompt diagnosis and management. *Streptococcus intermedius* is a recognized cause of brain abscesses, particularly in individuals with underlying conditions that compromise immune function. Sarcoidosis, a systemic granulomatous disease, may predispose patients to opportunistic infections due to immune dysregulation. To our knowledge, this is the first reported case of a cerebellar abscess caused by *S. intermedius* as the initial manifestation of sarcoidosis.

**Case presentation:**

A 45-year-old Caucasian male presented with progressively worsening occipital headache, dizziness, and gait instability. Neuroimaging revealed a right cerebellar abscess causing obstructive hydrocephalus. Emergency surgical intervention included external ventricular drainage and suboccipital craniectomy with abscess evacuation. Microbiological culture from the abscess confirmed *S. intermedius* as the causative organism. The patient’s past medical history included liver cirrhosis secondary to alcohol abuse, with 8 years of abstinence. Further diagnostic workup, including chest imaging and bronchoscopy, revealed bilateral pulmonary infiltrates. Histopathological analysis of a bronchial biopsy demonstrated non-caseating granulomas consistent with sarcoidosis. Immunological evaluation revealed T-cell lymphocytopenia, suggesting an immunocompromised state. The patient received targeted intravenous antibiotic therapy for 6 weeks, consisting of ceftriaxone and metronidazole for the *S. intermedius*-induced brain abscess, alongside cotrimoxazole for presumed *Pneumocystis jirovecii* pneumonia. At the 6-month follow-up, he remained neurologically intact, and repeat imaging confirmed complete resolution of the abscess.

**Conclusions:**

This case illustrates an unusual initial presentation of sarcoidosis manifesting as a cerebellar abscess caused by *S. intermedius*. Clinicians should maintain a high index of suspicion for underlying immunological conditions in patients presenting with atypical or severe infections. Early diagnosis and multidisciplinary management are essential for favorable outcomes in such complex cases.

## Background

Cerebellar abscesses are localized collections of pus within the cerebellum, typically resulting from infections that may originate from various sources, including contiguous infections of the ears or sinuses, as well as systemic infections. Cerebellar abscesses pose a life-threatening risk due to their potential to cause severe neurological deficits, elevate intracranial pressure, and result in death if not diagnosed and treated promptly [1, 2]. *Streptococcus intermedius*, a β-hemolytic Gram-positive coccus belonging to the anginosus group, is recognized as a common pathogen in the development of brain abscesses, including those of the cerebellum [3, 4]. This bacterium is typically part of the oral and gastrointestinal microbiota but can become etiologically significant under certain conditions, particularly in individuals with predisposing factors such as immunosuppression or mucosal disruption [5, 6].

The relationship between *S. intermedius* and sarcoidosis is clinically significant. Sarcoidosis is a systemic granulomatous disease that may induce an immunocompromised state, thereby increasing susceptibility to opportunistic pathogens such as *S. intermedius*. In patients with sarcoidosis, the presence of non-caseating granulomas may lead to peripheral CD4-positive lymphocytopenia, predisposing them to infections that would typically be controlled in immunocompetent individuals [7]. Furthermore, corticosteroid therapy for sarcoidosis can further elevate the risk of infections, including those caused by *S. intermedius* [8]. In summary, cerebellar abscesses caused by *S. intermedius* represent a serious medical condition, particularly in the context of sarcoidosis, where immune function may be impaired. Timely diagnosis and treatment are essential for effective management and improved patient outcomes [1, 4].

This case highlights the importance of recognizing atypical presentations of sarcoidosis and the potential impact of immunosuppression on susceptibility to bacterial infections. To our knowledge, a cerebellar abscess caused by *S. intermedius* as the initial manifestation of sarcoidosis has not been previously reported in the literature. This case is particularly noteworthy due to the significant diagnostic and therapeutic challenges it presented.

### Case presentation

A 45-year-old Caucasian man was admitted to the hospital in August 2024 with progressively worsening occipital headache, dizziness, and gait instability. A few days prior to admission, he had presented to the emergency department with similar complaints. His medical history was notable for liver cirrhosis secondary to alcohol abuse; however, he had been abstinent for 8 years. A cranial computed tomography (CT) scan demonstrated a hypodense lesion in the right cerebellar hemisphere. He was discharged with analgesic medication, but his condition deteriorated, and he returned to the emergency department 2 days later. On the day of admission, he was barely able to stand due to vertigo.

On physical examination, the patient was oriented but exhibited dysarthria, horizontal nystagmus, dysdiadochokinesis of the right extremity, dysmetria, and intention tremor. He was drowsy but promptly arousable. Vital signs in the emergency department were largely normal, except for mild tachycardia and tachypnea (temperature 37.1 °C, blood pressure 110/78 mmHg, respiratory rate 18 breaths/min, heart rate 112 beats/min).

### Investigations

**Fig. 1:**
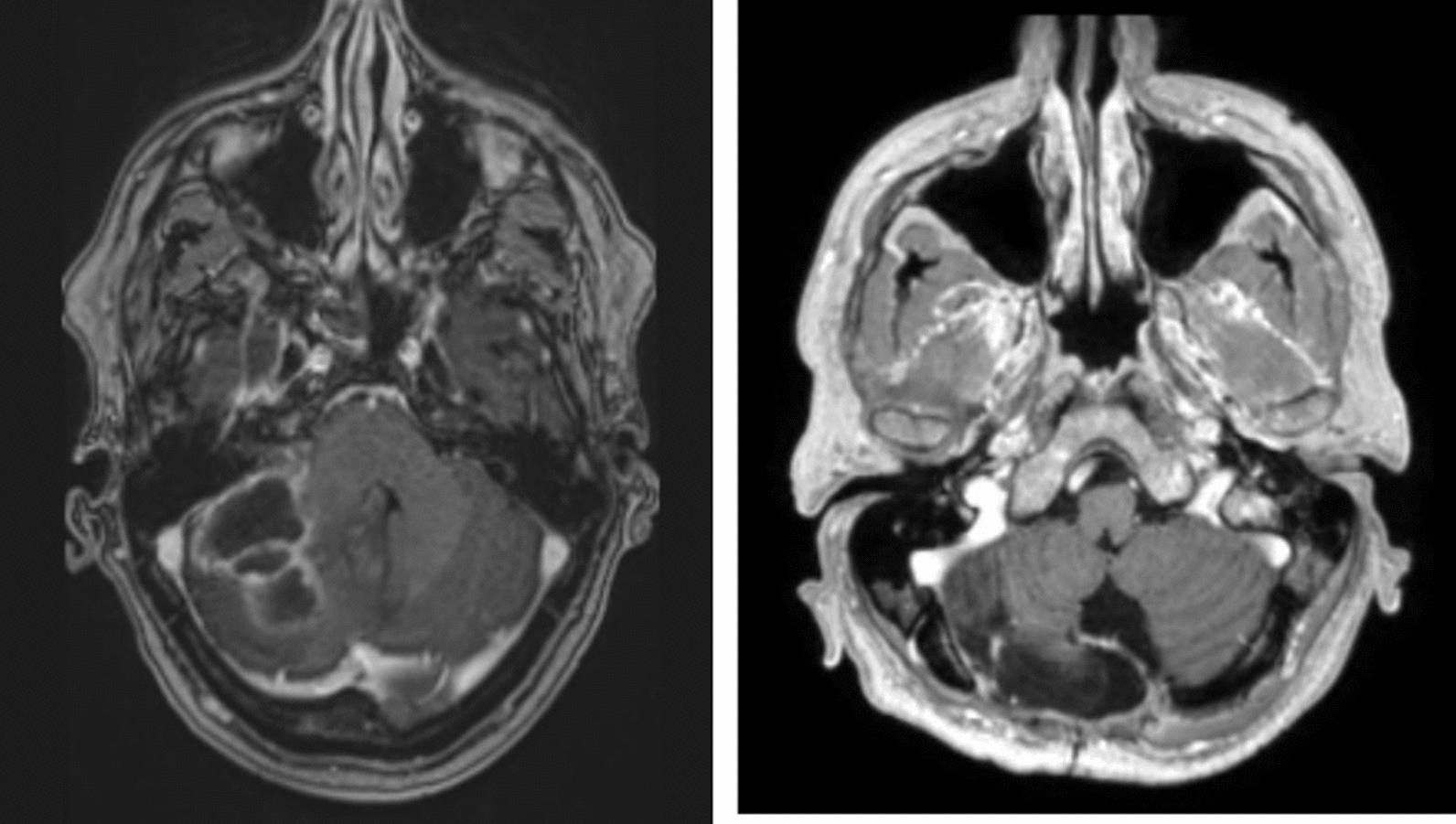
The initial magnetic resonance imaging with gadolinium enhancement revealed a lesion in the right cerebellar hemisphere, the follow-up magnetic resonance imaging with gadolinium enhancement after 6 months and after completion of antibiotic therapy showed no signs of residual lesions

Blood tests showed no leukocytosis, but revealed an elevated C-reactive protein level of 128 mg/L; other laboratory parameters were initially unremarkable. A repeat CT scan of the brain demonstrated progression of the hypodense lesion in the right cerebellar hemisphere, exerting pressure on the fourth ventricle and causing obstructive hydrocephalus. An emergency magnetic resonance imaging (MRI) scan subsequently revealed a gadolinium-enhancing lesion in the right cerebellar hemisphere with restricted diffusion and hyperintense signals on T2-weighted and fluid-attenuated inversion recovery (FLAIR) sequences, raising strong suspicion for a cerebellar abscess (Fig. 1).

### Treatment and outcome

Based on these findings, the patient was taken to the operating room with a preliminary diagnosis of a cerebellar abscess. A right-sided external ventricular drain (EVD) was placed to relieve obstructive hydrocephalus, followed by a suboccipital craniectomy with abscess evacuation without removal of the capsule. Postoperatively, the patient was admitted to the neurosurgical intensive care unit (ICU), and empirical broad-spectrum antibiotic therapy with vancomycin and meropenem was initiated.

In the search for the source of infection, oral and otorhinolaryngological examinations were unremarkable, with no history of recent dental procedures. Transesophageal echocardiography showed no evidence of valvular vegetations. However, chest radiography demonstrated bilateral atypical opacities, and subsequent chest CT revealed ground-glass opacities. Given these findings and the patient’s poor overall condition, extensive microbiological testing of serum and cerebrospinal fluid was performed for tuberculosis, human immunodeficiency virus (HIV), Epstein–Barr virus (EBV), cytomegalovirus (CMV), herpes simplex virus (HSV) types 1 and 2, *Cryptococcus* spp., *Coccidioides* spp., *Pneumocystis jirovecii*, *Treponema pallidum*, and *Toxoplasma gondii*, all of which returned negative results.

Flow cytometric immunophenotyping of peripheral blood demonstrated marked T-lymphocytopenia. CD3 + T lymphocytes were reduced to 44.6% (223/µL; reference range 600–2200/µL). CD4 + T-helper cells were significantly decreased at 20.2% (101/µL; reference range 400–1300/µL), while CD8 + cytotoxic T cells measured 23.0% (115/µL; reference range 120–900/µL), indicating borderline low absolute levels. The CD4/CD8 ratio was decreased to 0.88 (reference range 1.0–3.6). Natural killer cells were also reduced at 5.0% (reference range 6–29%), whereas CD19 + B lymphocytes were markedly elevated at 46.9% (reference range 7–23%). Overall, these findings demonstrated significant CD4-predominant T-cell lymphocytopenia with reduced NK cells, an inverted CD4/CD8 ratio, and relative B-cell expansion, consistent with an isolated T-lymphocyte deficiency as the underlying cause of immunosuppression. Blood and urine cultures showed no microbial growth. Bronchoalveolar lavage was positive for *P. jirovecii* DNA (52,250 copies/mL), prompting the addition of high-dose cotrimoxazole (1920 mg three times daily) to the antibiotic regimen.

Despite treatment, CRP levels and leukocyte counts continued to rise, and the patient developed high-grade fever. On the third hospital day, bacterial culture from intraoperative abscess aspirate grew *S. intermedius* without evidence of polymicrobial infection. According to susceptibility testing and microbiological recommendation, antibiotic therapy was adjusted to ceftriaxone and metronidazole for brain abscess-directed treatment, while cotrimoxazole was continued for presumed *Pneumocystis* pneumonia. A follow-up brain MRI on day five demonstrated significant perilesional edema and parenchymal swelling of the right cerebellum. The patient subsequently required intubation due to loss of protective reflexes. Bronchoscopy revealed purulent secretions. Histological examination of a transbronchial biopsy from the right upper lobe identified two sclerosed epithelioid cell granulomas—one within the bronchial wall and the other in dystelectatic lung parenchyma—findings highly compatible with sarcoidosis. The patient’s condition gradually stabilized, allowing extubation. The EVD was successfully weaned and removed, and the patient was transferred to the general ward for further monitoring before referral to the pulmonology department, where pulmonary sarcoidosis radiographic stage II was diagnosed.

The patient completed a 21-day course of cotrimoxazole in addition to 6 weeks of intravenous ceftriaxone and metronidazole. During hospitalization, he developed deep vein thrombosis secondary to immobility, which was treated with anticoagulation therapy. At the six-month outpatient follow-up, he exhibited no persistent neurological deficits, and MRI after completion of antibiotic therapy showed no residual diffusion restriction (Fig. 1). Repeat immunological evaluation three months after therapy demonstrated normalization of immune parameters with recovery of T-cell function.

## Discussion

Our case identifies *S. intermedius* as the causative pathogen of the cerebellar abscess, consistent with previous reports [1]. Comprehensive diagnostic evaluation additionally led to the diagnosis of sarcoidosis, highlighting the importance of considering underlying immunomodulatory diseases in patients presenting with unusual infections. Sarcoidosis is characterized by a paradoxical immune state in which granuloma formation may result in anergy and increased susceptibility to opportunistic infections [6]. Consequently, affected patients may be more vulnerable to pathogens such as *S. intermedius*, particularly in the setting of immunosuppressive therapy [6, 8].

*S. intermedius* is part of the normal oral and gastrointestinal microbiota but can become pathogenic under conditions such as immunosuppression. Although central nervous system involvement is uncommon, it has been reported in association with brain abscess formation [1, 3, 9]. Identification of the organism from the abscess aspirate supports its etiologic role in this case. Because infections often arise from mucosal disruption or hematogenous spread, evaluation for odontogenic, sinus, otogenic, gastrointestinal, or cardiac sources is recommended. When no primary focus is identified, this should be acknowledged as a limitation; however, the immune dysregulation associated with sarcoidosis provides a plausible predisposing factor for invasive disease.

Early recognition is essential to prevent complications and improve outcomes, as delayed diagnosis is associated with increased morbidity and mortality [1, 2]. Brain abscesses frequently present with nonspecific symptoms, and the classic triad of fever, headache, and focal neurological deficits is often absent, necessitating a high index of suspicion-particularly in immunocompromised patients [2]. Management requires prompt antimicrobial therapy guided by culture and susceptibility testing. Empirical regimens commonly include ceftriaxone combined with metronidazole (for covering anaerobes) with subsequent tailoring once microbiological results are available [2, 3]. In the present case, targeted therapy proved effective against the isolated pathogen.

Clinicians should also recognize that sarcoidosis is associated with infectious complications due to immune dysregulation and therapeutic immunosuppression, underscoring the need for multidisciplinary management. Bronchoalveolar lavage was positive for *P. jirovecii* DNA, prompting initiation of cotrimoxazole. However, molecular detection is highly sensitive and cannot reliably distinguish active pneumonia from colonization. Although bilateral pulmonary infiltrates and marked T-cell lymphocytopenia increased the likelihood of clinically relevant infection, the absence of typical respiratory features or supportive biomarkers would leave colonization as a possible alternative explanation. This diagnostic uncertainty should therefore be considered when interpreting the indication for PJP-directed therapy.

Infections in patients with sarcoidosis may mimic disease progression and delay diagnosis. Furthermore, brain abscesses can resemble metastatic lesions, emphasizing the importance of careful evaluation for infectious etiologies [1, 5]. Overall, the association between sarcoidosis and severe infections highlights the need for heightened clinical vigilance and coordinated, multidisciplinary patient care.

## Conclusion

This case highlights an unusual presentation of sarcoidosis, initially diagnosed through a cerebellar abscess caused by *S. intermedius*. A heightened awareness of potential underlying immunological disorders is crucial in patients with atypical infections.

## Data Availability

The original contributions presented in this study are included in the article. Further inquiries can be directed to the corresponding author.
